# Quality of Sleep among the Post Operative Patients at a Tertiary Care Hospital: An Observational Study

**DOI:** 10.31729/jnma.9159

**Published:** 2025-07-31

**Authors:** Narayan Mahotra, Lava Shrestha, Sonam Chaudhary, Tabassum Thakurai, Tara Bhandari

**Affiliations:** 1 Department of Clinical Physiology, Maharajgunj Medical Campus, Maharajgunj, Kathmandu, Nepal; 2 Maharajgunj Medical Campus, Maharajgunj, Kathmandu, Nepal.

**Keywords:** *post-operative*, *sleep quality*, *surgery*

## Abstract

**Introduction:**

A good sleep is important for recovery after stress like surgeries in patients. This study aims to study the quality of sleep postoperatively in our population to know the distribution of poor sleepers.

**Methods:**

A descriptive cross-sectional study was carried out from February, 2025 to April, 2025 after receiving ethical approval 081/082] in tertiary care hospital. The patients who were 18 years and above residing in the hospital after any surgery were included in the study. The general information of the patients was recorded followed by the responses in Pittsburgh Sleep Quality Index Questionnaire. Data was entered in Microsoft Excel 2016 and analysis was done using IBM SPSS Statistics version 16.0 and the descriptive statistics was used in the variables considered.

**Results:**

Out of 141 postoperative cases, only 133 participants were included for the analysis of Pittsburgh Sleep Quality Index questionnaire responses. The prevalence of poor sleepers was 92 (69.17%, 95% CI: 60.58%-76.89%). Severe difficulty was observed in the daytime dysfunction (27; 20.30%), sleep quality (17; 12.78%), and sleep latency (17; 12.78%) components of the Pittsburgh Sleep Quality Index. Among the poor sleepers, 67 (50.37%) were female, 40 (30.07%) were in the 40-59 years age group, and 86 (64.66%) were married. Of the total poor sleepers, 64 (48.12%) were within one week of the postoperative period.

**Conclusions:**

Poor sleep quality was prevalent after surgery, especially in the first week. It was more noticeable among females, middle-aged adults, and married individuals.

## INTRODUCTION

Sleep is an important physiological process which helps to conserve body energy for restoring the activities. There can be changes in body functions, mental illness, and other health problems due to sleep disturbances. Sleep deprivation is also associated with immunosuppression1 along with neurological dysfunction and psychological changes such as increased irritability, anxiety, and depressive symptoms.^2-4^ This will lead to increased co-morbidity, mortality, health care costs, and poor quality of life among poor sleepers.^5^ It is important to know the sleep quality among the post operative patients also as it can lead to delay in recovery and feeling of unwell after surgery. The postoperative sleep disturbance is reported to be high in different studies with an incidence between 16% and 67.3%. ^6-8^ There is lack of similar studies in Nepalese patients. So, this research project aimed to know the quality of sleep among post operative patients in our population. This article thus represents the distribution of poor sleepers among such population.

## METHODS

This study was a questionnaire-based cross-sectional study that was carried out among the admitted patients of Tribhuvan University Teaching Hospital Institute of Medicine, Maharajgunj, Kathmandu, Nepal. This funded research project had the objective to find the sleep quality among post-operative patients and the risk factors associated with it, in which the primary analysis regarding the distribution of sleep quality among these participants is only represented in this study. The study was carried out from February 2025 to April 2025 after getting the ethical clearance from the Institutional Review Committee of IOM with the reference number: 460(6-11) E2 081/082. The data was collected among the post-operative patients who were 18 years and above who had surgeries and were residing in surgical wards. The participants were enrolled after the informed consent by a convenient sampling method. The cases admitted to the postoperative ward or Intensive Care Units, cases of severe illness requiring frequent vital sign monitoring, maternity cases, patients with multiple surgeries in the past 3 months, patients with history of any primary psychiatric illnesses and the cases of uremic encephalopathy, hepatic encephalopathy, central nervous system infection, toxic and substance intoxication were excluded.^8^ The sample size of 141 was calculated with the help of prevalence of postoperative sleep disturbance of 90% prevalence and with margin of error of 0.05.^9^ The study tool consisted of the Pittsburgh sleep quality index (Full version) for assessing the sleep quality among the operated patients as it is a validated tool for assessing the sleep quality in similar studies and other studies as well in Nepalese population.^10,11^ This self-report questionnaire has 19 questions with seven different subcategories. These subcategories included sleep quality, latency, duration, and disturbance; habitual sleep efficiency; use of sleep medications, and daytime dysfunction. The questions belong to seven different subcategories of this questionnaire. The method of scoring of the Pittsburgh Sleep Quality Index (PSQI) parameter was based on a 0 to 3 scale, where 3 reflects the extremely negative response on the Likert scale. The scores of few of the questions had to be calculated to get the score of certain subcategories. The sum of the scores from all seven subcategories produces a global score. The global score can range from 0 to 21, with higher scores associated with a poorer quality of sleep. The PSQI score of >6 points were used to distinguish good from poor sleepers, with a PSQI score of >6 points indicated as sleep disturbance ^12^

The data collection sheet contained all the details of the patient to know about desired variable as discussed further. The research team members were well trained to ask the questions, filling the responses and recording the informations as needed in the study. Data was entered in Microsoft Excel 2016. The statistical analysis was done with using SPSS Statistics for Windows, version 16.0 (SPSS Inc., Chicago, Ill., USA). There were few responses received from the patients residing more than 1 months and such data were excluded during the calculation of poor sleep quality. The age of participants was classified as per the division of adulthood as young adults ≤39 years, 40-59 years as middle adults and ≥60 years as old aged.^13^ The gender and marital status were classified as male, female and married, unmarried respectively. Similarly, the post operative day was calculated on the basis of days between the date of surgery and date of data collection and the operational classification included within 1^st^ week after surgery, within the 1^st^ and 2^nd^ week after surgery, between 15^th^ day to end of 1^st^ month, between 1^st^ to 2^nd^ month after surgery and more than 2 months after surgery. The descriptive statistics were used to find the prevalence of poor sleepers among the post-surgical patients in these different categories of the variables considered.

## RESULTS

Among the 141 total patients, responses from 133 patients within one month of the postoperative period were included. Of these, 62 (46.61%) were aged less than 40 years. The mean age was 42.33 ± 14.93 years. There were 95 (71.42%) female and 121 (90.97%) married participants. Responses were obtained from 96 (68.08%) patients during the first postoperative week (Table 1).

**Table 1:** Distribution of participants according to demographic (n=133).

Variables	n(%)
Age (years)	
18-39	62(46.61)
40-59	54(40.60)
≥60	17(12.78)
Gender	
Male	38(28.57)
Female	95(71.42)
Marital status	
Married	121(90.97)
Unmarried	12(9.02)
Post operative day (after Surgery)	
1st week	96(68.08)
1st week - 2nd week	18(12.76)
2nd week -1st month	19(13.47)

The global PSQI score showed that 92 (69.17%, 95% CI: 60.58%-76.89%) participants were poor sleepers post operatively. Participants with severe difficulty in daytime dysfunction were 27 (20.30%), severe difficulty in sleep quality and sleep latency, each with 17 (12.78%), (Table 2).

**Table 2:** Pittsburgh Sleep Quality Index (PSQI) component responses among the participants (n=133).

PSQI components	Responses in Likert Scale (3: severe difficulty; 1: No difficulty)			
	3	2	1	0
Sleep Quality	17	45	54	17
n(%)	(12.78)	(33.83)	(40.60)	(12.78)
Sleep Latency	17	46	36	34
n(%)	(12.78)	(34.58)	(27.06)	(25.56)
Sleep Duration	15	16	40	62
n(%)	(11.27)	(12.03)	(30.07)	(46.61)
Sleep Disturbance	3	42	87	1
n(%)	(2.25)	(31.57)	(65.41)	(0.75)
Sleep Efficiency	14	2	13	104
n(%)	(10.52)	(1.50)	(9.77)	(78.19)
Use of Medicine	3	4	5	121
n(%)	(2.25)	(3.00)	(3.75)	(90.97)
Daytime Dysfunction	27	68	16	22
n(%)	(20.30)	(51.12)	(12.03%)	(16.54)

**Table 3:** Distribution of poor sleepers among the participants (n=133).

Variables	Poor Sleepers n(%)	Normal Sleepers n(%)
Age (years)		
≤39(62)	38(28.57)	24(18.04)
40-59 (54)	40(30.07)	14(10.52)
≥60 (17)	14(10.52)	3(2.25)
Gender		
Male (38)	25(18.79)	13(9.77)
Female (95)	67(50.37)	28(21.05)
Marital Status		
Married (121)	86(64.66)	35(26.31)
Unmarried (12)	6(4.51)	6(4.51)
Post Operative Day Group (after Surgery)		
1^st^ week (96)	64(48.12)	32(24.06)
1^st^ week - 2^nd^ week (18)	12(9.02)	6(4.51)
2^nd^ week -1^st^ month (19)	16(12.03)	3(2.25)

The distribution of poor sleepers among postoperative patients aged 40-59 years was 40 (30.07%). Female poor sleepers were 67 (50.37%) and male poor sleepers were 25 (18.79%). Poor sleepers among married participants were 86 (64.66%) and among unmarried participants were 6 (4.51%). Poor sleepers within the 1^st^ week after surgery were 64 (48.12%) and those between the 2nd week and 1^st^ month after surgery were 16 (12.03%) (Table 3).

## DISCUSSION

Sleep is an important physiological process that preserve body energy and restores body processes. Sleep deprivation in post operative phase promotes the development of catabolic state which can have an adverse effect in the recovery processes after surgery.^14,15^ The global score of Pittsburgh Sleep Quality Index questionnaire showed that the maximum participants involved in this study had poor sleep quality (69.00%). In these responses, the maximum difficulty was seen as presence of daytime dysfunction (20.30%) followed by difficulty in sleep quality (12.78%) and sleep latency (12.78%). Similar study conducted in Ethiopia had showed the prevalence of poor sleepers to be 64.90%^10^ while in India, the prevalence of poor sleepers post operatively when compared with pre-operative condition was 33.3%.^8^ The post operative sleep disturbance was high on different studies which was inadequately managed in different areas of the world with an incidence between 16% and 67.3%.^10^

This Indian study had also showed that the distribution of poor sleepers post operatively was maximum in participants more than 60 years age (53.25%) followed by the cases in between 36-60 years and least were in those who were <36 years. The increasing age could be related with decreased capacity to cope with surgical stress mentally and physically to cause increase post-surgical sleep disturbance among this group. Aging is also associated with the structural changes in sleep which makes elderly more prone to face difficulty to adjust their sleep to environmental changes.^16,17^ However, the finding contradicts with the Ethiopian study which has found the adult surgical patients between 25-54 years were 15.2 times more likely to develop postoperative sleep disturbance in comparison with those extreme ages.^10^ This study also has found the percentage distribution of poor sleepers were more in between 40-59 years of age followed by 18 to 39 years age group.

This Ethiopian study has also found the distribution of poor sleepers among male patients (58.5%) was comparatively more than female. It had reported that the male gender patients were 4.81 times more likely to develop the poor post operative sleep quality.^10^ The male patients being addictive to substances like alcohol and smoking disturbing the patient’s mode of sleep among them was reported in literature.^18^ This is in contrast with our study with relatively more female being poor sleepers post operatively than male. The increase female participants with comparatively increased poor sleep quality were found in other study as well.^8^ The increase number of female participants had reported worse sleep quality, increase post inflammatory response, increase pain level than men in different group of surgical patients as well.^19^ The gender-wise unequal distribution of participants in this study could be the reason for more poor sleepers post-operatively among female. Similarly, the post operative sleep disturbance was seen more among the married patients in this study which is supported by other studies as well.^12^ The family and responsibilities stress could be responsible for this more sleep disturbances among married people which needs further exploration. The post operative sleep disturbance was more common among the patients who had surgery in past 1 week in this study. The finding is similar with the study which shows decreasing trend of post operative sleep disturbances with increasing duration after surgery among the orthopaedic cases.^9^ The initial post operative days are accompanied with pain and other distress which needs to be explore further. Research has also shown that the incidence of sleep disturbances can reach over 90% within 2 days after surgery.^20^

It has been reported that the post operative deterioration of sleep quality is multifactorial in origin which can include the surgical inflammatory response, the severity of surgical trauma, pain, anxiety, the use of anesthetics and environmental factors such as nocturnal noise and light levels.^21^ There will be further exploration of the risk factors associated with poor sleep quality with more analysis of data collected in this research project. Also, the limitation of this whole research project is the inclusion of post operative patients after different types of surgery which can alter the type of anaesthesia, trauma, recovery, hospital stay and associated mental stress among the participants which can influence the sleep quality.^21^ The study if had conducted in similar group of participants with the comparison of pre-operative sleep quality could have provide a better insight about the impact of that particular surgery in sleep quality so that a strong step could be implemented by the stakeholder for improving the well-being of such patients. Therefore, the future research with pre-operative baseline assessments or comparisons group can robustly establish causality.

The polysomnography of sleep among post operative patients have found the decrease in total sleep time by upto 80% with fragmented sleep and either a decrease or complete loss of REM and N3 sleep.^22-24^ Thus, the sleep cycle among the post operative patients can also be further explored in future in our population.

## CONCLUSIONS

The study found that most postoperative patients experienced poor sleep quality, particularly in the areas of daytime dysfunction, sleep quality, and sleep latency. Sleep disturbances were more evident among females, middle-aged adults and married individuals.

## Data Availability

The data are available from the corresponding author upon reasonable request.
